# Estriol strongly inhibits DNCB-induced contact dermatitis: role of antigen-specific antibodies in pathogenesis

**DOI:** 10.1530/EC-14-0080

**Published:** 2014-09-16

**Authors:** Elizabeth Yan Zhang, Bao-Ting Zhu

**Affiliations:** 1 Department of Pharmacology, Toxicology and Therapeutics, School of Medicine, University of Kansas Medical Center, Room 4061 of KLSIC Building, 2146 West 39th Street, Kansas City, Kansas, 66160, USA; 2 Department of Biology, South University of Science and Technology of China, Shenzhen, Guangdong, 518055, China

**Keywords:** estrogens, estriol, estrone, 17β-estradiol, DNCB-induced contact dermatitis, delayed type hypersensitivity, skin hypersensitivity, antigen-specific antibody, humoral immune responses

## Abstract

The endogenous estrogens are important modulators of the immune system and its functions. However, their effects are rather complex and many aspects have not been studied. In this study, we used the 1-chloro-2,4-dinitrobenzene (DNCB)-induced contact dermatitis as a disease model and investigated the effect of estriol (E_3_), along with two other estrogens, 17β-estradiol and estrone, on the pathogenesis of contact hypersensitivity. A series of parameters, such as ear swelling, skin inflammation, antigen-specific immunoglobulins, and lymphocyte compositions in peripheral lymphoid organs, were evaluated in mice following development of contact dermatitis. We found that administration of all three estrogens elicited strong inhibition of DNCB-induced dermatitis, while E_3_ exerted the strongest suppressive effect. Administration of E_3_ alleviated dermatitis, and this effect was accompanied by decreases in serum DNCB-specific immunoglobulins, such as IgA, IgG1, IgG2a, and IgG2b. Besides, treatment with E_3_ reduced B cell population, especially IgG-producing cells in the peripheral lymphoid organs following the induction of dermatitis. These observations consistently suggest that the antibody (Ab)-mediated humoral immune reactions play a critical role in the pathogenesis of DNCB-induced contact dermatitis. The results from this study demonstrate, for the first time, that estrogen administration has a strong suppressive effect on the pathogenesis of contact dermatitis. These findings offer important insights concerning the pathogenic role of antigen-specific Abs in contact dermatitis and the treatment of chemical-induced, Ab-mediated skin hypersensitivity reactions in humans.

## Introduction

Many earlier studies have shown that estrogens are important modulators of immune system functions. It was demonstrated that although physiological levels of estrogens are immunostimulatory, high or super-physiological levels of estrogens, such as those observed during human pregnancy, are immunosuppressive (1). For example, 17β-estradiol (E_2_) and estriol (E_3_) at suitable doses can inhibit the development of experimental autoimmune encephalomyelitis (EAE) (2, 3, 4) and collagen-induced arthritis in murine models (3), and E_2_ can also suppress inflammatory response in castrated male mice (5). Besides, certain E_2_ derivatives were found to have a beneficial effect on inflammatory bowel disease in a murine model (6).

The hapten-induced contact dermatitis, also called contact hypersensitivity (CHS) or delayed type hypersensitivity (DTH), was initially considered as a predominant T cell-mediated immune response (7, 8, 9, 10, 11, 12). Lately, it was reported that B-1 cells or NK cells are also involved in the mediation of this immune response (9, 13, 14, 15). We have recently shown that B cells and the formation of hapten-specific antibodies (Abs) play a critical role in the pathogenesis of hapten-induced CHS (16).

While a few earlier studies have suggested that E_2_ can inhibit DTH reactions (17), there was only one earlier study published some 30 years ago that examined the effect of E_3_ on DTH reactions in mouse footpads against injected sheep red blood cells (SRBC) (18). In that study, however, a conclusion could not be reached because E_3_ was found to have a suppressive effect against a low dose of the SRBC antigen, whereas promoting the reaction when higher doses of the antigen were used. In this study, we sought to investigate the modulating effect of E_3_, an unique estrogen that is predominantly produced in large quantity during human pregnancy (19, 20), on the allergic contact dermatitis induced by topical application of 1-chloro-2,4-dinitrobenzene (DNCB), and its effect was compared with two other common estrogens, E_2_ and estrone (E_1_).

## Materials and methods

### Chemicals and reagents

DNCB (99% purity) was purchased from Acros Organics (Fair Lawn, NJ, USA). E_1_, E_2_, E_3_, cholesterol, dexamethasone (Dex), Tween-20, albumin from chicken egg white (ovalbumin, OVA, 98% purity), and 1,2-phenylenediamine (OPD, 99.5% purity) were obtained from Sigma–Aldrich. Fluorochrome-conjugated Abs were purchased from BD Biosciences (San Jose, CA, USA), eBioscience (San Diego, CA, USA), or Biolegend (San Diego, CA, USA). Other reagents used in this study were obtained from Fisher Scientific (Pittsburgh, PA, USA).

### DNCB-induced contact dermatitis in mice

Experimental protocols involving the use of live animals were approved by the Institutional Animal Care and Use Committees (IACUC) of the University of South Carolina (Columbia, SC, USA) and the University of Kansas Medical Center (Kansas City, KS, USA). The 6- to 8-week-old male *Balb/c* mice with the average body weight of 18 g were obtained from Harlan Laboratories (Houston, TX, USA). After arrival, they were allowed to acclimatize for a week before used in experimentation. The animals were housed under controlled conditions of temperature (22 °C) and photoperiod (12 h light:12 h darkness cycle), and they were allowed free access to food and water throughout the experiment.

**Figure 1:**
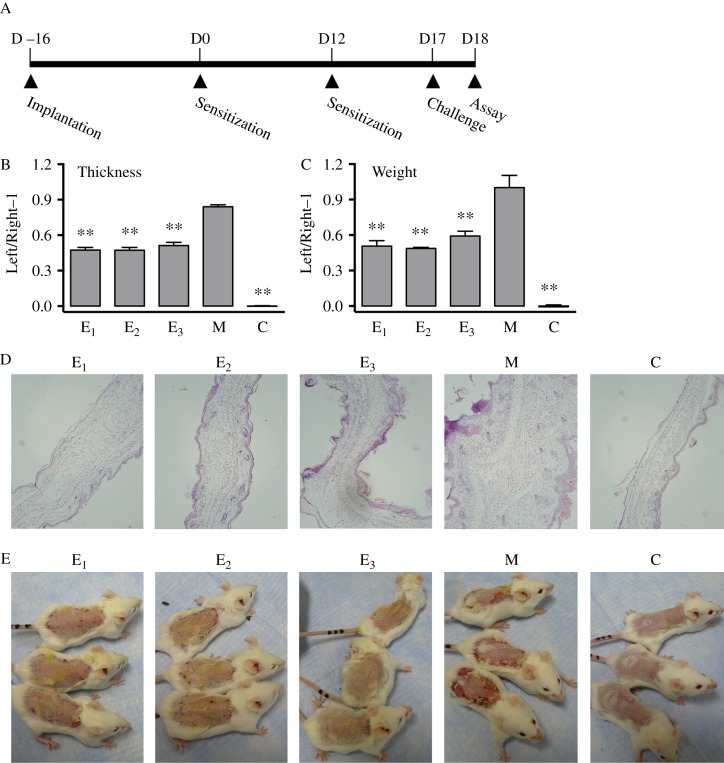
Administration of estrogen significantly inhibits DNCB-induced contact dermatitis. (A) The experimental schedule used in this study. Each mouse was implanted with a pellet containing an estrogen or vehicle under the back skin on day −16, and sensitized by painting 100 μl of 2% DNCB in ethanol or vehicle on the shaved back skin on day 0 and day 12. The mouse was then challenged by painting 20 μl of 2% DNCB in ethanol on the left ear twice with a 60-min interval on day 17, and assays were carried out the next day. (B and C) Ear swelling index was based on the increase in thickness (B) and weight (C) from the DNCB-challenged left ear to vehicle-challenged right ear using the following formula: value (thickness or weight) of left ear/right ear−1. (D) Histological changes in H/E-stained tissue sections. (E) The severity of skin inflammation on the back of mice at 8 days after first sensitization with DNCB. It should be noted that in the data shown in this figure, mice labeled as group ‘E_3_’ received E_3_+DNCB; mice labeled as group ‘E_2_’ received E_2_+DNCB; mice labeled as group ‘E_1_’ received E_1_+DNCB; mice labeled as group ‘M’ received a vehicle pellet+DNCB; and mice labeled as group ‘C’ received vehicles only. *n*=6 for each group. **P<0.01 vs the group of mice treated with DNCB alone ‘M’.

To induce contact dermatitis, the animals were first sensitized by painting 100 μl of 2% DNCB in ethanol onto the shaved back skin twice with a 12-day interval. Five days later, 20 μl DNCB was painted on the left ear twice with a 60-min interval (Fig. 1A). Twenty-four hours later, the ear swelling was evaluated by measuring the differences in the thickness (with an engineer's micrometer) and the weight of a small round piece cut out by using a sharp clamp between the right and left ears. The control animals were painted with ethanol alone on their shaved backs and left ears. There were six to eight mice in each group.

### Administration of estrogens and Dex

Ten milligrams of E_1_, E_2_, or E_3_ and 15 mg cholesterol were mixed thoroughly and then manually pressed by the same person by applying consistent pressure using a Pellet Presser (Parr Instrument Company, Moline, IL, USA) to produce pellet with a total weight of 25 mg. The vehicle pellet (containing only 25 mg cholesterol) was prepared in the same manner. Each pellet was implanted surgically under the back skin of each animal 16 days before the start of the DNCB treatment. The control animals received the pellets that contained 25 mg cholesterol alone. One day before the DNCB treatment, each mouse in the Dex-treated group received an i.m. injection of Dex at 10 mg/kg b.w. once every 2 days until the end of the experiment when the animals were killed.

### Histopathological analysis

The formalin-fixed, paraffin-embedded ear tissues were sectioned at 5-μM thickness, and the sections were stained with hematoxylin and eosin (H/E). The pictures were taken using a light microscope at a 100× magnification.

### Weight index

The mouse body weight was measured right before the animals were killed. The spleen and thymus were removed and weighted. The weight index refers to the weight of an organ (mg) divided by the total body weight (g).

### Flow cytometry analysis

Immediately after the inguinal lymph nodes (LNs), spleens, and thymus were isolated, they were ground and the cells were strained to obtain the single cell suspensions. The cell quantity was determined by using a hematocytometer. After incubation with the Ab conjugated with fluorochrome followed by washing twice with FACS buffer (2% FBS in PBS), the samples were fixed with 2% paraformaldehyde in PBS overnight and measured on the flow cytometer, and the data were analyzed using the Flowjo software (Tree Star, Inc., Ashland, OR, USA).

### Measurement of serum Ab levels

The dinitrophenyl–OVA conjugate was prepared by stirring 1% OVA in the sodium borate buffer (0.05 M, pH 9.4) at 4 °C. DNCB powder (0.01 mol) was gradually added to the solution followed by dialysis against the sodium borate buffer. The solution was centrifuged at 300 ***g*** for 5 min. The supernatants were sequentially dialyzed against distilled water at 4 °C. The conjugates were then lyophilized and stored at −80 °C until use. The measurement of DNCB-specific Abs and total Abs was carried out as previously reported (16).

### Statistical analysis

Data are presented as mean±s.d. and were analyzed using a one-way ANOVA or two-way ANOVA and a multiple comparisons *post hoc* analysis (Dunnett's method) to test the difference between the DNCB treatment only group and the other groups.

## Results

### Inhibition of DNCB-induced contact dermatitis by estrogens

To test the role of estrogens (E_3_, E_2_, and E_1_) on the pathogenesis of contact dermatitis, the animals received s.c. implantation of a 25 mg pellet containing 10 mg E_3_, E_2_, or E_1_ to provide a sustained release of the estrogens. Sixteen days after pellet implantation, the animals were sensitized with DNCB and followed by a second sensitization 12 days later. DNCB challenge reaction was given 5 days after second sensitization and measurements were made 24 h later (Fig. 1A).

Treatment of animals with E_3_, E_2_, or E_1_ attenuated DNCB-induced ear swelling, based on changes in ear thickness and wet weight (Fig. 1B and C). Histopathological analysis showed that treatment with DNCB alone induced severe inflammatory infiltration, vascular congestion, and moderate edema in ear dermis (Fig. 1D). In comparison, skins of mice co-treated with an estrogen displayed only mild cellular infiltration and vasodilation without marked edema (Fig. 1D). After exposure to DNCB alone for 8 days, the animals began to develop strong skin hypersensitivity reactions in the treated areas (Fig. 1E). This observation is consistent with our earlier observations (16), i.e., dermatitis began to appear on sensitized back skin 8 days after initial exposure to DNCB, and the scar formation was usually very severe and would last for several days. However, the degree of skin inflammation in animals co-treated with E_3_, E_2_, or E_1_ was markedly reduced compared with animals treated with only DNCB. Notably, the degree of skin inflammation in E_3_-treated mice was least severe, and E_3_ alleviated the hypersensitivity reaction in the back skin to a level almost comparable with the animals treated with vehicle only (without DNCB) (Fig. 1E).

### Effects of estrogens on body weight, organ weight index, and cell numbers in the lymphoid organs of mice that develop DNCB-induced contact dermatitis

**Figure 2:**
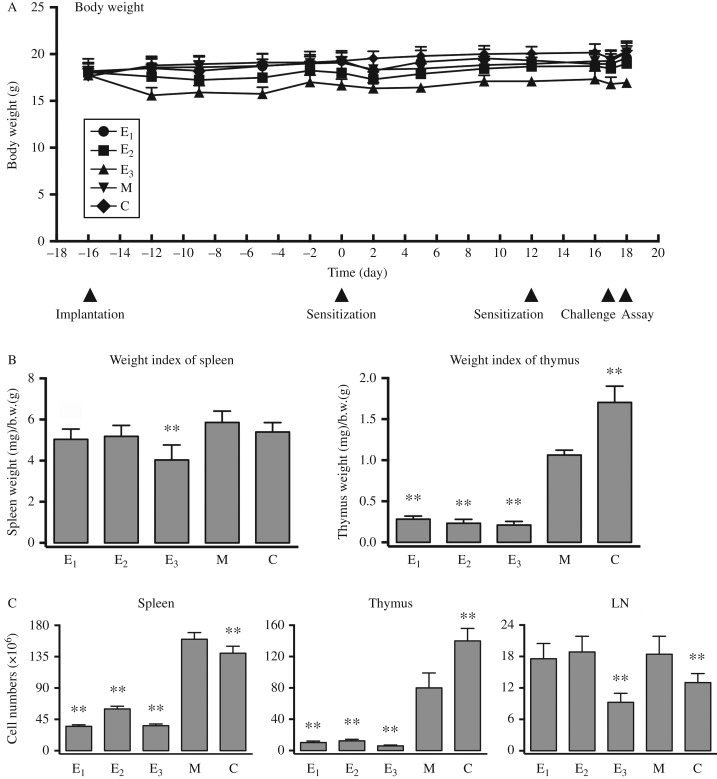
Effect of estrogen treatment on body weight, organ weight indices, and cell numbers in lymphoid organs from mice with DNCB-induced dermatitis. (A) The change in body weight during the experiment is shown. (B) On day 18, the weight of different organs was measured. The organ weight indices (left panel: spleen; right panel: thymus) were calculated using this formula: organ weight (mg)/body weight (g). (C) On day 18, the cell numbers of lymphoid organs were determined by a hemocytometer (left panel: spleen; middle panel: thymus; right panel: peripheral lymph nodes). Refer to Fig. 1 for the meaning of the group labels. *n*=6 for each group. **P<0.01 vs the group of mice treated with DNCB alone ‘M’.

To determine the toxicity of the chemical agents administered, change in the body weight during the experiment as well as the changes in spleen and thymus weight indices at the end of the experiment was measured. After the first sensitization with DNCB on the back skin, the animals exhibited a slight decrease (<6%) in body weight compared with vehicle-treated animals. However, after implantation of an E_3_ pellet, the body weight of each mouse rapidly decreased. E_3_ caused a 12% body weight reduction in 4–5 days. The body weight of E_3_-treated mice increased gradually afterwards. The average plasma concentrations of free E_3_ 5 days after pellet implantation were found to be at 3.6 ng/ml (12.5 nM) (data not shown; referred in (21)). In comparison, treatment of animals with E_1_ or E_2_ produced smaller reductions in their body weight (Fig. 2A).

DNCB treatment did not alter spleen weight index significantly, but decreased thymus weight index by ∼40% compared with vehicle-treated animals. While the animals treated with E_3_+DNCB had a 30% reduction in their spleen weight index, animals treated with E_2_ or E_1_+DNCB did not have significant reduction in their spleen weight index (Fig. 2B). In the thymus, treatment of E_3_+DNCB drastically reduced its weight index by up to 80% compared with animals treated with DNCB alone, and this reduction was ∼90% compared with vehicle-treated mice. Co-administration of E_2_ or E_1_+DNCB also caused huge decrease in thymus weight index (Fig. 2B).

DNCB caused a small increase in the number of splenocytes. However, in mice treated with DNCB+an estrogen (E_3_, E_2_, or E_1_), the splenocyte numbers were significantly reduced compared with DNCB treatment alone, and these numbers were even lower than the animals treated with vehicle alone (Fig. 2C).

In the thymus, DNCB treatment reduced the thymocyte numbers by ∼40%, which is consistent with the change in thymus weight (Fig. 2C). Addition of an estrogen (E_3_, E_2_, or E_1_) to DNCB-treated mice further reduced thymocyte numbers to ∼15% of the numbers seen in animals treated with DNCB alone (Fig. 2C). It is evident that among the three estrogens tested, E_3_ produced the strongest reduction in thymocyte population (Fig. 2C).

DNCB treatment increased the cell numbers in peripheral LNs by ∼40% over vehicle treatment. While mice receiving E_1_ or E_2_ in addition to DNCB did not alter LN cell numbers compared with DNCB alone, co-treatment with E_3_ reduced LN cell numbers by up to 50% compared with DNCB alone (Fig. 2C).

### Treatment with estrogens significantly suppressed the DNCB-specific Abs in mice that develop DNCB-induced contact dermatitis

**Figure 3:**
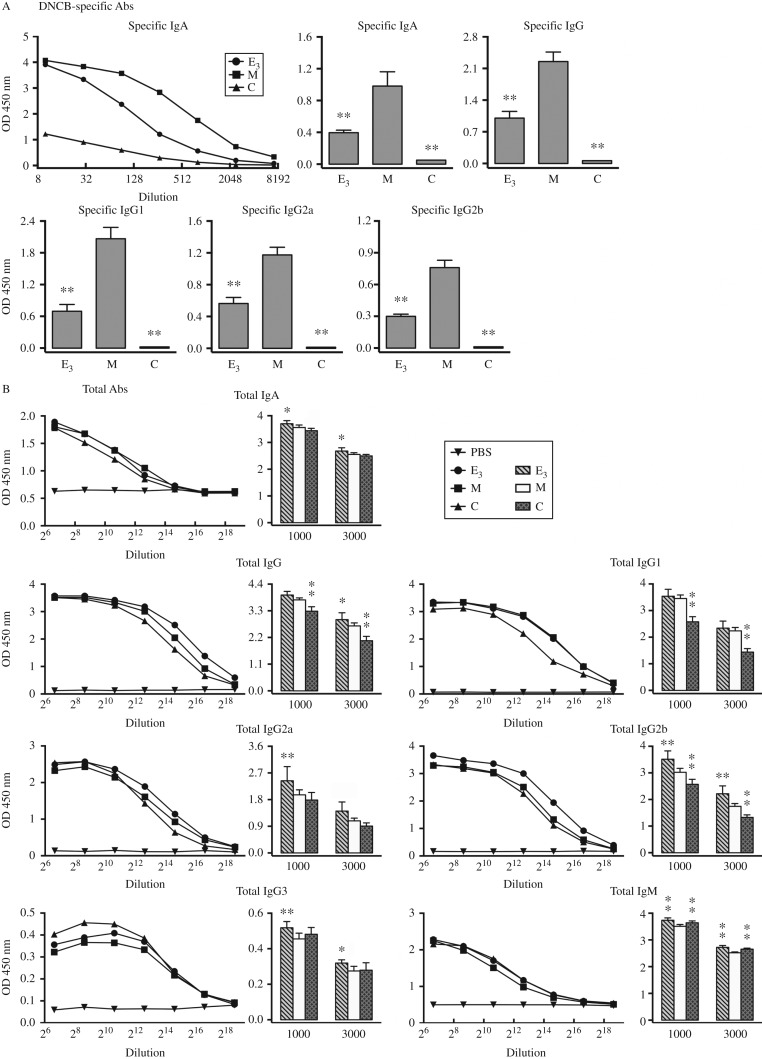
Administration of E_3_ strongly suppresses DNCB-specific Abs in the sera of mice with DNCB-induced dermatitis. (A) Serum levels of DNCB-specific Abs on day 18 following DNCB treatment. Top left panel: serial dilution (1:10, 30, 90, 270, 810, 2430, and 7290) of DNCB-specific IgA. The average value from two mice in each group was shown. Top middle, right, and bottom panels: mean±s.d. showing serum levels of DNCB-specific IgA, IgG, IgG1, IgG2a, and IgG2b at 1:600 dilution. *n*=6 for each group *P<0.05; **P<0.01 vs the group of mice treated with DNCB alone ‘M’. (B) Serum levels of total Abs on day 18 following DNCB treatment. Line plots: average value of sera from two mice in each group showing the serial dilution (1:100, 400, 1600, 6400, 25 600, 102 400, and 409 600) of total IgA, IgG, IgG1, IgG2a, IgG2b, IgG3, and IgM. PBS was also diluted with assay buffer as negative control. Bar plots: mean±s.d. showing serum levels of total IgA, IgG, IgG1, IgG2a, IgG2b, IgG3, and IgM at the dilution of 1:1000 and 1:3000. Referred in Fig. 1 for the meaning of the group labels. *n*=6 for each group *P<0.05; **P<0.01 vs the group of mice treated with DNCB alone ‘M’.

We previously reported that the antigen-specific Abs play an indispensable role in the development of antigen-induced CHS (16). Therefore, we investigated in this study whether the suppressive effect of estrogens on the pathogenesis of DNCB-induced CHS is associated with the levels of DNCB-specific Abs. Because E_3_, among the three estrogens tested, has the strongest inhibition of CHS (Fig. 1E), we presented only the data regarding the modulating effect on DNCB-specific Abs. The suppression of DNCB-induced skin hypersensitivity by E_3_ (as presented in Fig. 1B, C, D, and E) was found to be accompanied by marked reductions (≥50%) in DNCB-specific serum Abs (e.g., specific IgA, IgG, IgG1, IgG2a, and IgG2b) compared with animals treated with DNCB alone (Fig. 3A).

In contrast to the induction of DNCB-specific Abs, DNCB treatment did not significantly change the serum levels of total IgA, IgG2a, and IgG3, only slightly reduced the levels of total IgM, but increased the levels of total IgG, particularly IgG1 and IgG2b (Fig. 3B). The effects of E_3_ on serum levels of total Abs were more complex. Treatment of E_3_+DNCB did not produce change in serum levels of total IgG1 as compared with the animals treated with DNCB alone, but it increased the serum levels of total IgA, IgG, IgG2a, IgG2b, IgG3, and IgM. It is of note that E_3_ treatment brought the total IgG3 and IgM levels nearly equal to the levels observed in vehicle-treated control animals (Fig. 3B).

### Effect of estrogens on the lymphocyte composition in peripheral lymphoid organs of mice with DNCB-induced contact dermatitis

Contact dermatitis is usually treated by topical or systemic application of glucocorticoids, such as Dex (11, 22). We have reported earlier that administration of Dex inhibited DNCB-induced CHS by suppressing the production of DNCB-specific Abs (16). In this study, we found that E_3_ exerted a similar suppressive effect on DNCB-induced CHS, and this effect was accompanied by a significant decrease in the production of DNCB-specific Abs. Hence, we included Dex as a positive control for suppressing the pathological development of contact dermatitis in some of the additional analyses which sought to compare whether E_3_ exerts similar effects as Dex on peripheral lymphocyte depletion (23).

**Figure 4:**
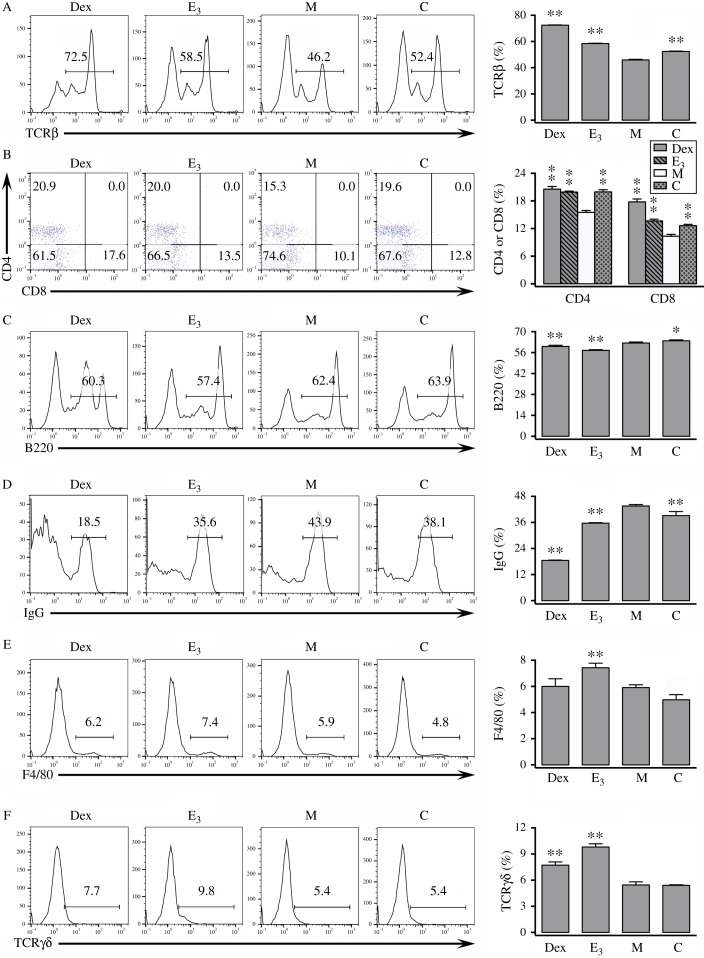
Effect of E_3_ treatment on the lymphocyte composition in spleen. Splenocytes from mice with DNCB-induced dermatitis or control mice were stained with various fluorochrome-conjugated antibodies and assayed for different cell surface markers. Left panels: representative histograms or dot plots. Right panels: summarized bar plots. *n*=3 for each group. *P<0.05; **P<0.01 vs the group of mice treated with DNCB alone ‘M’. Refer to Fig. 1 for the meaning of the group labels. In addition, mice labeled as group ‘Dex’ received Dex+DNCB. (A) Percentages of splenocytes expressing TCRβ. (B) Percentages of splenocytes expressing CD4 or CD8. (C) Percentages of splenocytes expressing B220. (D) Percentages of splenocytes expressing IgG. (E) Percentages of splenocytes expressing F4/80. (F) Percentages of splenocytes expressing TCRγδ.

In splenocytes, DNCB treatment reduced the percentage of T cells (e.g., TCRαβ^+^, CD4^+^, and CD8^+^ cells) compared with the control animals (Fig. 4A and B), but the percentage of splenic B cells (e.g., B220^+^ and IgG^+^ cells) were not similarly changed by the treatment (Fig. 4C and D). DNCB treatment did not have marked changes in the percentages of splenic macrophages (F4/80^+^) or TCRγδ^+^ cells (Fig. 4E and F) (16). Co-administration of E_3_ or Dex increased the percentage of splenic T cells (TCRαβ^+^, CD4^+^, and CD8^+^ cells) (Fig. 4A and B), but reduced the percentage of splenic B220^+^ and IgG^+^ cells (Fig. 4C and D). While Dex had a stronger effect than E_3_ in the induction of TCRαβ^+^ cells and the suppression of IgG^+^ cells, both of them increased the levels of TCRγδ^+^ cells, with E_3_ having a stronger effect (Fig. 4A and D). In addition, E_3_ significantly increased the percentage of splenic macrophages whereas Dex did not show a similar effect (Fig. 4E and F).

**Figure 5:**
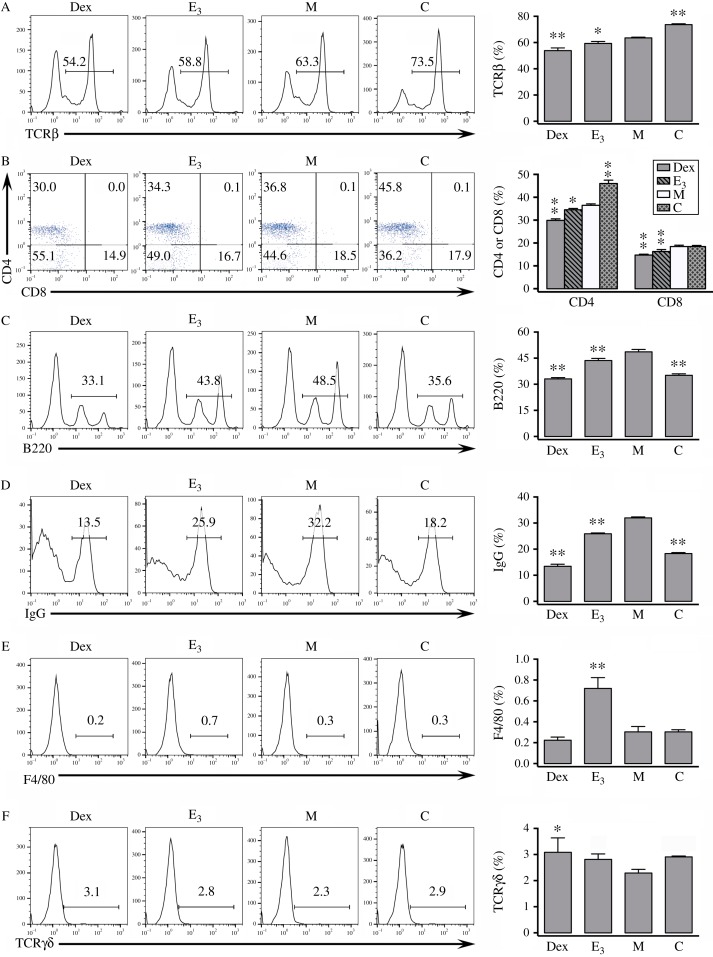
Effect of E_3_ treatment on the lymphocyte composition in lymph nodes. Cells of peripheral lymph nodes from mice with DNCB-induced dermatitis or control mice were stained with various fluorochrome-conjugated antibodies and assayed for different cell surface markers. Left panels: representative histograms or dot plots. Right panels: summarized bar plot. *n*=3 for each group. *P<0.05; **P<0.01 vs the group of mice treated with DNCB alone ‘M’. Refer to Fig. 4 for the meaning of the group labels. (A) Percentages of lymph node cells expressing TCRβ. (B) Percentages of lymph node cells expressing CD4 or CD8. (C) Percentages of lymph node cells expressing B220. (D) Percentages of lymph node cells expressing IgG. (E) Percentages of lymph node cells expressing F4/80. (F) Percentages of lymph node cells expressing TCRγδ.

In peripheral LNs, treatment with DNCB decreased the percentage of T cells (TCRαβ^+^ and CD4^+^) (Fig. 5A and B), but increased the percentage of B cells (B220^+^ and IgG^+^) (Fig. 5C and D). In general, DNCB treatment inhibited T cells, especially T_H_ cells, but promoted the production of B cells in peripheral LNs. Compared with DNCB treatment alone, co-administration of E_3_ or Dex+DNCB decreased the percentage of LN T cells (TCRαβ^+^, CD4^+^, and CD8^+^ cells) (Fig. 5A and B), and also decreased the percentage of B220^+^ and IgG^+^ cells (Fig. 5C and D). The animals treated with Dex had the lowest percentage of LN B220^+^ and IgG^+^ cells, which was even lower than the vehicle group (Fig. 5C and D). Treatment of E_3_, but not Dex, increased the percentage of macrophage (F4/80^+^) compared with the DNCB treatment alone (Fig. 5E). However, Dex, but not E_3_, increased the percentage of TCRγδ^+^ cells (Fig. 5F). Therefore, E_3_ and Dex displayed rather different effects on lymphocyte composition in peripheral lymphoid organs of mice that developed DNCB-induced contact dermatitis.

The composition of thymocytes was found to be similar in mice treated with DNCB alone or vehicle alone (data not shown), thereby suggesting that the changes in thymocytes appear to be less important in the pathogenesis of DNCB-induced contact dermatitis.

## Discussion

The results of our present study showed that the DNCB-induced contact dermatitis is associated with a strong inhibition of peripheral T cells (Figs 4A, B and 5A, B) and thymocytes number (Fig. 2C). Co-administration of E_3_ produced a further drastic inhibition of LN T cells and thymocytes. This effect of E_3_ has led to the suggestion that its rescue mechanism in DNCB-induced contact dermatitis likely is not mediated through changes in T cells and their functions. By contrast, treatment of mice with DNCB increased the B cell composition in peripheral lymphoid organs (Figs 4D and 5C, D), accompanied by increases in DNCB-specific Abs (Fig. 3A). Co-treatment with E_3_ brought these parameters to the levels close to vehicle-treated animals. It was quite amazing to observe that administration of E_3_, E_1_, or E_2_, strongly alleviates DNCB-induced contact dermatitis by reducing inflammatory reactions. It has been reported that estrogen can regulate humoral immunity by modulating B cell development and function, such as Ab production (24). This information is in line with the notion that E_3_ may inhibit CHS via reduction of B cell composition and function, and ultimately, the production of antigen-specific Abs.

It is of interest to note that we found in this study that E_3_ has a stronger therapeutic effect than E_2_ (a far potent and efficacious endogenous estrogen) in an antigen-induced contact dermatitis model. Similar observations with E_3_ have also been reported earlier by us and others. For instance, it has been shown that E_3_ given at doses that can reach blood levels commonly seen during late stages of human pregnancy can produce a longer effect in delaying EAE onset than did E_2_ (3). Our recent studies (21, 25) have also shown that E_3_ has rather distinct functions from E_2_ in the regulation of certain immune system functions, including the production of antigen-specific Abs and some splenocyte functions.

One of the possible explanations for the different effects of the three endogenous estrogens tested in this study may depend on their differential binding affinities for the estrogen receptors (ERs). E_2_ has high and similar binding affinity for both ERα (ESR1) and Erβ (ESR2), and its binding affinities for both ERs are significantly higher than those of E_3_ and E_1_ (26). However, E_1_ has a preferential binding affinity for ERα over ERβ, and E_3_ has a preferential binding affinity for ERβ over ERα (26). ERα and ERβ are expressed in immune cells of both human and mice (27, 28), and thus it is possible that estrogens may have a direct effect on the immune system. This hypothesis is supported by some of the earlier studies. For instance, it was reported that E_2_ can directly act on CD4^+^/CD25^−^ T cells via ER (29), which then directly interacts with NFκB to regulate the production of inflammatory cytokines (30, 31). However, it is also possible that estrogen may first act on other types of cells rather than lymphocytes to alter the production of regulatory factors and/or the activation of other cell types, which then further act on the immune system to modulate its functions. This possibility cannot be ruled out at present.

As mentioned earlier, E_3_ is a rather unique hormone in humans, and it is predominantly produced in large quantity during late pregnancy (19, 20). It has been reported that during human pregnancy, the circulating levels of total E_3_ drastically increased from the base levels (usually below 50 nM) to nearly 700 nM at the late stages (32). In our studies, 4–5 days after pellet implantation, at which time the toxicity of estrogen treatment based on body weight change reaches peak level, the average plasma levels of free E_3_ were found to be at 12.5 nM (21), a level that is within the physiological range. As the body weight loss reaching maximum (12%) at 4–5 days after estrogen pellet implantation, it quickly returned close to control level, suggesting that the animals recovered from the initial adverse effect of estrogen treatment. Like estrogen, treatment of Dex, which is a common therapeutic agent for contact dermatitis, also causes some weight loss (33, 34). Therefore, the observed levels of transient body weight change following estrogen treatment may be viewed as an acceptable adverse effect. In this study, it should also be mentioned that the pellets used in this study are of a sustained release type, which lasts for several months after implantation (35) and thereby provides a good method of administering estrogens for studying their effect on DNCB-induced CHS.

In the literature, there is actually no report concerning a role of pregnancy hormones in allergic contact dermatitis. Nevertheless, relevance between sex hormones and contact dermatitis can be deduced on the basis of some of the earlier studies. First, estrogens are known to promote skin elasticity and improve wound healing (36). Second, it was reported that skin response to allergens is markedly stronger at follicular phase than during ovulation (37, 38, 39). Third, Bonamonte *et al*. reported that significantly fewer healthy women display intense responses to nickel-induced skin allergy during ovulation where endogenous estrogen levels are very high compared with luteal phase (17, 40). Lastly, some studies have suggested that ovulatory hormonal factors may act to suppress DTH reactions (17, 41, 42). The findings of our present preclinical study, which demonstrate that administration of estrogens can strongly suppress the pathogenesis of allergic contact dermatitis, provide a good mechanistic explanation for these intriguing earlier clinical observations. In addition, these findings may offer a useful strategy for effectively alleviating the clinical symptoms of allergic human contact dermatitis.
